# Glossopharyngeal neuralgia after SARS-CoV-2 infection: A case report

**DOI:** 10.4317/jced.63439

**Published:** 2025-11-30

**Authors:** Hector Martin Vargas Cornejo, Cesar Augusto Jiménez Prado, Manuel Fernando Guillén Galarza

**Affiliations:** 1 DDS, MSc (Stomatology). National University of Trujillo. Faculty of Stomatology. Trujillo, Peru. https://orcid.org/0000-0002-1815-9605; 2 DDS, PhD (Stomatology). National University of Trujillo. Faculty of Stomatology. Trujillo, Peru. https://orcid.org/0000-0002-9444-9188; 3 DDS, PhD (Stomatology). National University of Trujillo. Faculty of Stomatology. Trujillo, Peru. https://orcid.org/0000-0002-9684-9898

## Abstract

Glossopharyngeal neuralgia (GN) is a rare neuropathic disorder characterized by sudden, unilateral, electric shock-like pain in the areas innervated by the glossopharyngeal nerve. Its diagnosis is frequently delayed because of its clinical overlap with odontogenic and otorhinolaryngological conditions. In the context of the COVID-19 pandemic, different cranial neuropathies have been reported, suggesting possible post-infectious mechanisms. We describe the case of a 54-year-old male dentist, without relevant medical history, who developed recurrent episodes of intense pain in the right pharynx and base of tongue after confirmed SARS-CoV-2 infection. Symptoms were triggered by swallowing, coughing, and salivary stimulation, reaching maximum intensity on the visual analogue scale (EVA 10/10). Brain and neck magnetic resonance imaging revealed no structural abnormalities. Treatment with carbamazepine (600 mg/day) partially reduced frequency and severity of attacks, while pregabalin (300 mg/day) showed no benefit. This case highlights the need to consider SARS-CoV-2 infection as a potential trigger of GN, underscores the importance of recent infectious history in the differential diagnosis, and emphasizes the relevance of early pharmacological management in clinical improvement.

## Introduction

Glossopharyngeal neuralgia (GN) is an uncommon neuropathic disorder characterized by paroxysmal episodes of severe, unilateral, lancinating or electric shock-like pain in the areas innervated by the ninth cranial nerve, including the base of the tongue, tonsil, pharynx, and ear. These episodes are frequently triggered by speaking, swallowing, or coughing (1 - 3). GN accounts for only 0.2-1.3% of all cranial neuralgias, with an estimated incidence of 0.7 cases per 100,000 inhabitants per year (2 - 4).

Its etiology may be idiopathic or secondary to various conditions, such as neurovascular compression, cerebellopontine angle tumors, anatomical malformations, infections, Eagle's syndrome, or demyelinating lesions (1 , 2 , 5). Diagnosis is based on the ICHD-3 criteria, which include brief, stabbing, unilateral pain with abrupt onset and resolution, without interictal neurological deficits, and requires the exclusion of other causes through clinical history, physical examination, and imaging studies (4). Due to its similarity with other otorhinolaryngological or dental conditions, diagnosis is often delayed, leading to multiple consultations and ineffective treatments (6).

First-line treatment is pharmacological, with carbamazepine or oxcarbazepine as drugs of choice. In refractory cases, alternatives such as gabapentinoids, nerve blocks, or microvascular decompression may be considered (4). In rare circumstances, GN may coexist with other hyperactive dysfunction syndromes, such as trigeminal neuralgia, which further complicates diagnosis (6).

In the context of the COVID-19 pandemic, several neurological manifestations have been documented, including cranial neuropathies attributable to viral neuroinvasion or immune-mediated dysfunction. Among them, cases of post-COVID-19 GN have been reported, expanding the etiological spectrum of this entity and posing new challenges for diagnosis and management (5).

Glossopharyngeal neuralgia is a rare entity, difficult to diagnose, and usually associated with structural causes such as neurovascular compression. However, during the COVID-19 pandemic, neurological manifestations related to SARS-CoV-2 infection, including cranial neuropathies, have been described. In this regard, cranial nerves may be affected during the disease, supporting the possibility that the virus may act as a trigger for cranial neuropathies, including GN (7).

Reporting this case of post-COVID-19 GN is relevant because it broadens the recognized etiological spectrum, highlights the need to consider infectious history in the differential diagnosis, and provides clinical evidence to optimize the management of this rare neuralgia. The aim of this report is to describe the clinical presentation of a case of glossopharyngeal neuralgia following SARS-CoV-2 infection.

## Case Report

A 54-year-old mestizo male, dentist by profession, with no relevant medical history or systemic diseases, presented in April 2021 with odynophagia localized in the posteroinferior region of the tongue and right pharynx. The pain was atypical, pulsatile, and circumscribed to an area of approximately 1 mm², with an initial intensity of 50/100 on the visual analogue scale (VAS). It did not improve with a course of sodium naproxen 550 mg every 8 hours for three days. Subsequently, the patient developed myalgias, fatigue, dysgeusia, hyporexia, anosmia, and headache. On April 24, 2021, he was clinically diagnosed with SARS-CoV-2 infection, presenting with severe cough and hypoxemia (minimum oxygen saturation of 89%). He received symptomatic and antibiotic management (azithromycin), with slow recovery in an outpatient setting and persistence of cough for several weeks.

The case was characterized by a semiannual periodicity of neuralgic crises lasting around 20 days, with average remission periods of 5 months.

The first episode, corresponding to October 2021, began with a prodromal symptom of continuous, intense, lancinating pain, well-circumscribed to a single point in the right submandibular region around the tonsil. The pain was described by the patient with an intensity of 7/10, and within the following 48 hours, he experienced continuous symptoms for 20 days, with 5-6 daily neuralgic crises of an average duration of 4 minutes each. Every crisis typically consisted of 5 sudden attacks with the characteristics detailed in Table 1.Table 1

The second episode occurred in April 2022, beginning with prodromal pain similar to the first, followed by 21 days of more intense and frequent symptoms. This coincided with mild COVID-19, where coughing acted as the main trigger. During this period, manipulation of the external ear canal provoked paroxysmal pain (EVA 10/10), confirming glossopharyngeal nerve involvement. Otolaryngology evaluation showed no pathology.

**Figure 1:**
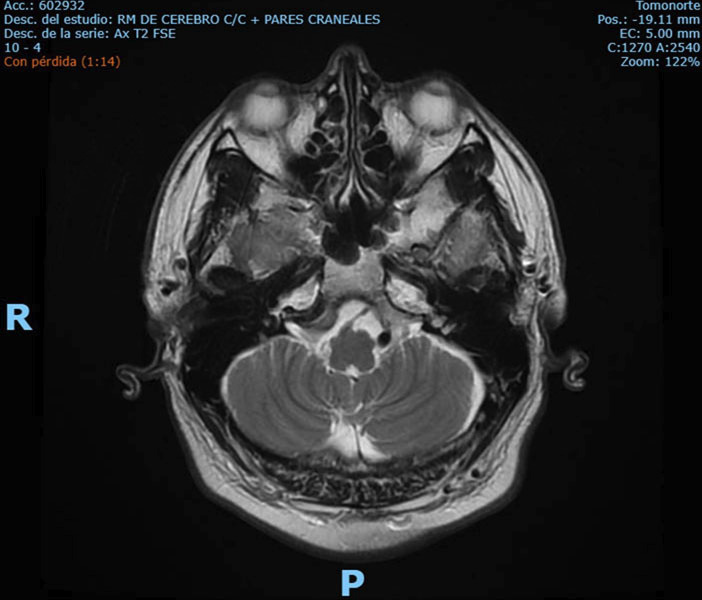
Brain MRI in axial T2 sequence showing skull base and cerebellopontine angle structures without evident lesions.

For diagnostic purposes, Neurology and Internal Medicine requested complementary studies. Brain MRI with contrast, cranial nerve sequences, maxillofacial and cervical imaging, and cervical ultrasound revealed no abnormalities (Fig. 1).

1

Based on clinical features and imaging, a diagnosis of glossopharyngeal neuralgia likely secondary to COVID-19 was established.

Carbamazepine (200-1000 mg/day) achieved ~50% reduction in attack frequency and intensity, while pregabalin was ineffective. Semiannual recurrences persisted until 2023, but in 2024 symptoms diminished to brief painful 'clicks.' The last episode was in September 2024, and at follow-up in September 2025 (age 58) the patient reported one year free of neuralgic crises, reflecting favorable long-term evolution.

## Discussion

This clinical case provides evidence of a possible temporal association between SARS-CoV-2 infection and glossopharyngeal neuralgia (GN), a rare condition whose diagnosis is often delayed due to its similarity with dental or otolaryngological pathologies (1 , 5). The temporal onset of neuralgia following confirmed episodes of COVID-19, together with the absence of structural findings on imaging studies, points to a functional or post-infectious inflammatory mechanism, thereby expanding the traditionally recognized etiological spectrum of this cranial neuropathy.

The clinical relevance of this observation lies in the need for healthcare professionals to consider a history of viral infection, particularly SARS-CoV-2, in the differential diagnosis of cranial neuralgias. Recognizing this association may help avoid unnecessary treatments, guide the early selection of pharmacological therapy, and improve patient quality of life through a more targeted approach.

From a pathophysiological perspective, the literature describes several mechanisms that may explain the relationship between COVID-19 and cranial neuropathies, including systemic hypoxemia, immune dysfunction, neuroinflammation, and direct viral invasion of the nervous system (8). The involvement of multiple cranial nerves in the context of COVID-19 reinforces this hypothesis and suggests that the virus may act as a trigger for neuralgias in predisposed patients (8).

**Figure 2:**
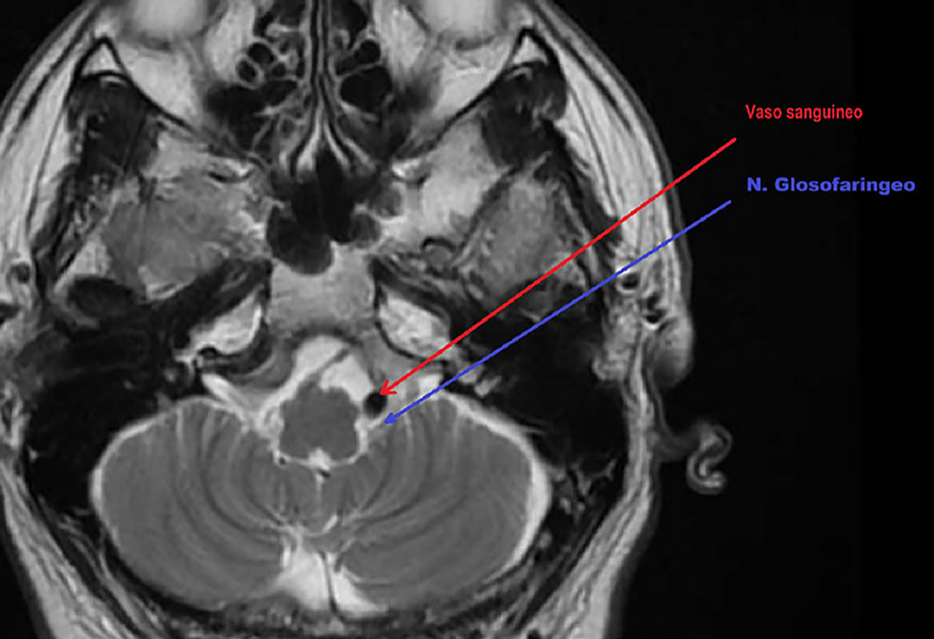
MRI reconstruction showing the anatomical relationship between the glossopharyngeal nerve (blue arrow) and an adjacent blood vessel (red arrow). Colored arrows were digitally added for illustration purposes.

Additionally, neuroimaging played an essential role in ruling out structural causes of GN. In our case, MRI assessment (Fig. 2) revealed the anatomical relationship between the glossopharyngeal nerve and adjacent vascular structures, although no definitive compression was identified, reinforcing its diagnostic value.

2

The novelty of this report lies in the long-term follow-up, which demonstrated gradual spontaneous improvement after recurrent post-COVID episodes, a finding rarely documented in GN. Nevertheless, as a single case report, it lacks generalizability and additional laboratory or neurophysiological confirmation. Future multicenter studies are required to determine prevalence, risk factors, and long-term outcomes of post-COVID neuralgias.

## Figures and Tables

**Table 1:** Clinical characteristics of glossopharyngeal neuralgia episodes in a 54-year-old male patient.

Variable	Description
Onset	Sudden, fulgurant
Triggering and aggravating factors	Salivary stimulation by food and/or drinks, swallowing, routine movements upon standing up, swallow, yawn, sneeze or cough.
Relieving factors	None
Pain type	Stabbing, electric-like
Location and radiation	Submandibular region, posterior tongue in relation to the tonsils.
Severity	EVA 10/10
Duration	1–2 seconds per attack, with ~30-second remission until the next attack, repeatedly

Legend: EVA = Visual Analogue Scale for pain intensity.

## Data Availability

The datasets used and/or analyzed during the current study are available from the corresponding author.
